# Diagnostic challenges in breast carcinosarcoma: a case report of a rare and aggressive tumor

**DOI:** 10.3389/fmed.2025.1633085

**Published:** 2025-08-06

**Authors:** Yiwen Li, Caiyun Bai, Yuye Ma, Zhimin Fan, Xiaozhen Wang, Fengjiang Qu

**Affiliations:** ^1^Department of Breast Surgery, General Surgery Center, The First Hospital of Jilin University, Changchun, China; ^2^Department of Emergency Surgery, The First Hospital of Jilin University, Changchun, China

**Keywords:** breast cancer, carcinosarcoma, preoperative diagnostic challenges, phyllodes tumor, metaplastic carcinoma

## Abstract

**Background and objectives:**

Breast carcinosarcoma is rare. This article reports a case of diagnosis and treatment to enhance clinicians’ understanding and experience in managing this disease.

**Methods:**

A 44-year-old patient with breast carcinosarcoma treated at the Breast Surgery Department of the First Hospital of Jilin University is reported, highlighting the challenges in the diagnostic process.

**Results:**

Postoperative pathology confirmed carcinosarcoma. The patient underwent mastectomy, chemotherapy, and radiotherapy, with no recurrence during follow-up.

**Conclusion:**

The nonspecific clinical manifestations of breast carcinosarcoma make preoperative diagnosis challenging, requiring a comprehensive diagnostic approach.

## Introduction

Breast carcinosarcoma represents an exceptionally rare form of malignancy, comprising less than 0.2% of all breast cancers. This neoplasm is distinguished by its unique composition, featuring both epithelial and mesenchymal tumor elements, which significantly complicates preoperative diagnostic efforts. Herein, we present a comprehensive case report of a 44-year-old female patient diagnosed with breast carcinosarcoma who underwent treatment at the Department of Breast Surgery, First Hospital of Jilin University. The purpose of this report is to augment the understanding and clinical acumen of surgeons in the diagnosis and management of this uncommon disease entity.

## Materials and methods

**Figure 1:**
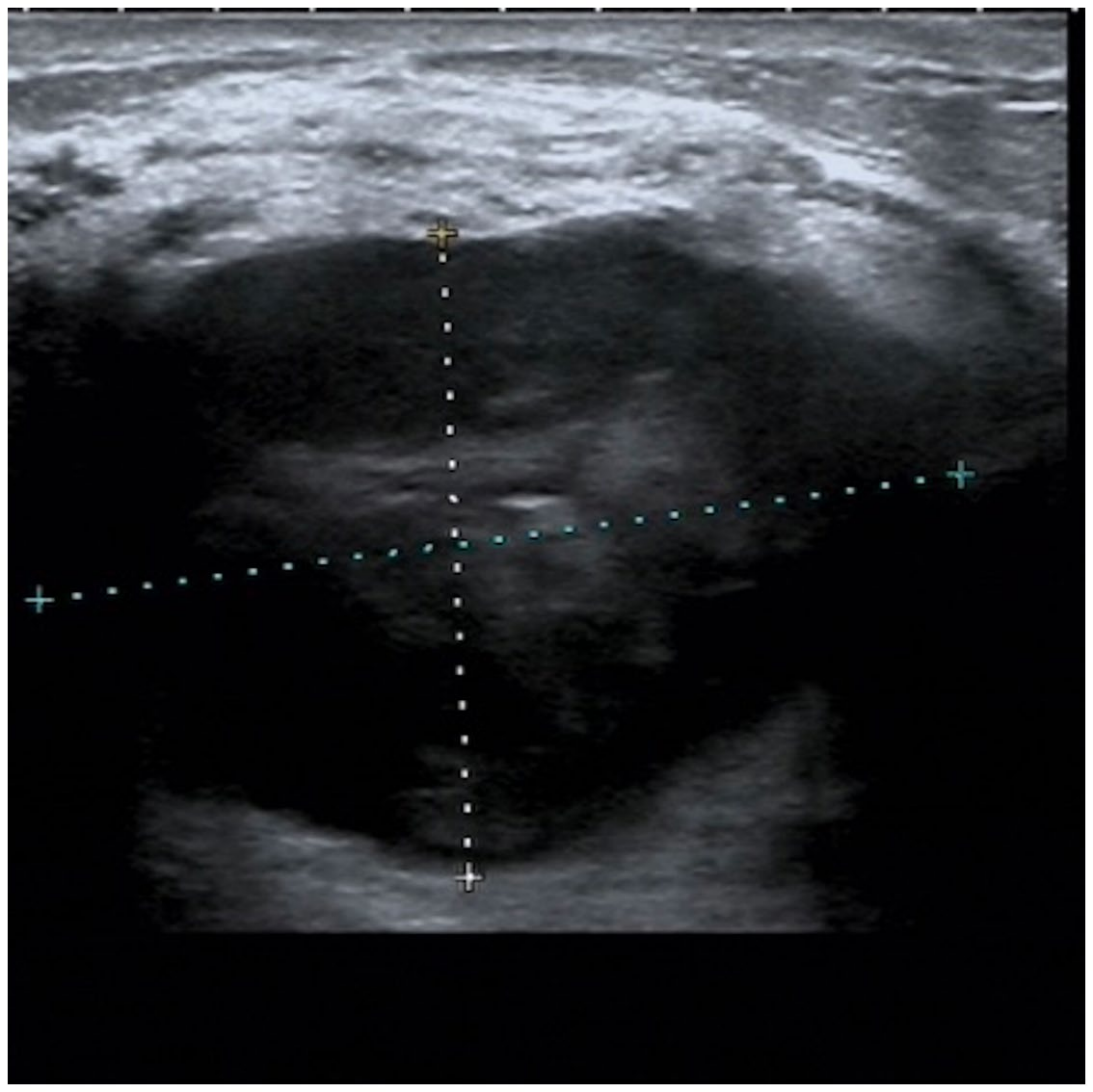
Ultrasound from June 20, 2022. A hypoechoic mass measuring 48.8*34.5 mm was observed in the upper outer quadrant of the right breast, with unclear boundaries and a relatively regular shape.

A 44-year-old woman presented with a 3 cm mass in her right breast, accompanied by bloody nipple discharge. Throughout her illness, she experienced intermittent fever spikes up to 38.2°C, which subsided with the use of antipyretic medication. The patient had no significant medical history, no history of childbirth, and no family history of breast disease. On physical examination, a 5.0 × 3.0 cm irregular mass was palpated in the outer upper quadrant of the right breast. The mass had indistinct borders and limited mobility but was not significantly tender to palpation. Ultrasound on June 20, 2022 imaging, revealed a 48.8 × 34.5 mm hypoechoic mass with poorly defined margins and rich vascularity in the outer upper quadrant of the right breast (Figure 1).

**Figure 2:**
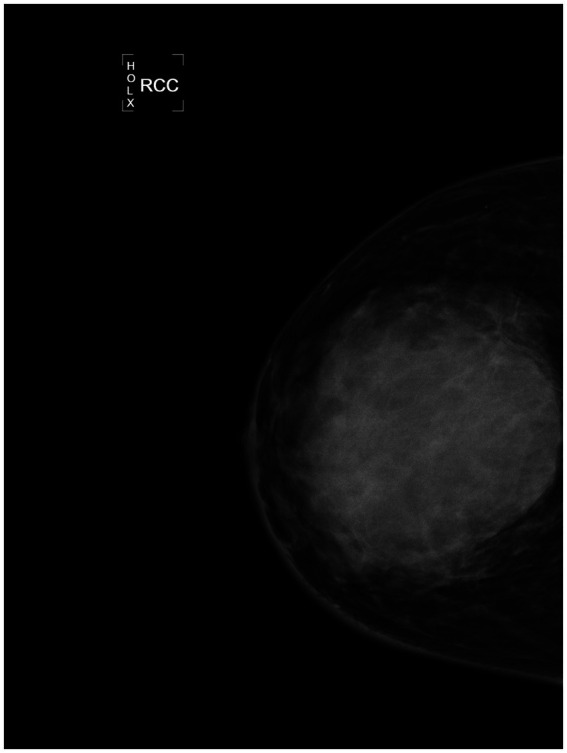
RCC of mammography image of the affected side from August 26, 2022. An irregular mass measuring 9.1*8.6 cm is visible in the upper quadrant, showing high density and blurred margins.

**Figure 3:**
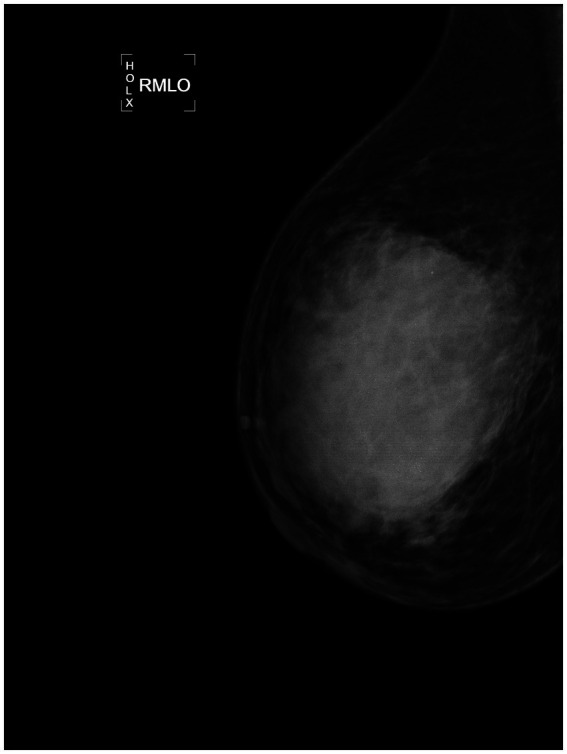
RMLO of mammography image of the affected side from August 26, 2022. An irregular mass measuring 9.1*8.6 cm is visible in the upper quadrant, showing high density and blurred margins.

Additionally, enlarged lymph nodes with cortical thickening were observed in the right axillary region. Mammographic findings on August 26, 2022, demonstrated a more significant 9.1 × 8.6 cm irregular, high-density mass with indistinct borders in the upper quadrant of the right breast (Figures 2, 3). The imaging characteristics suggested a potentially aggressive lesion, warranting further diagnostic evaluation.

**Figure 4:**
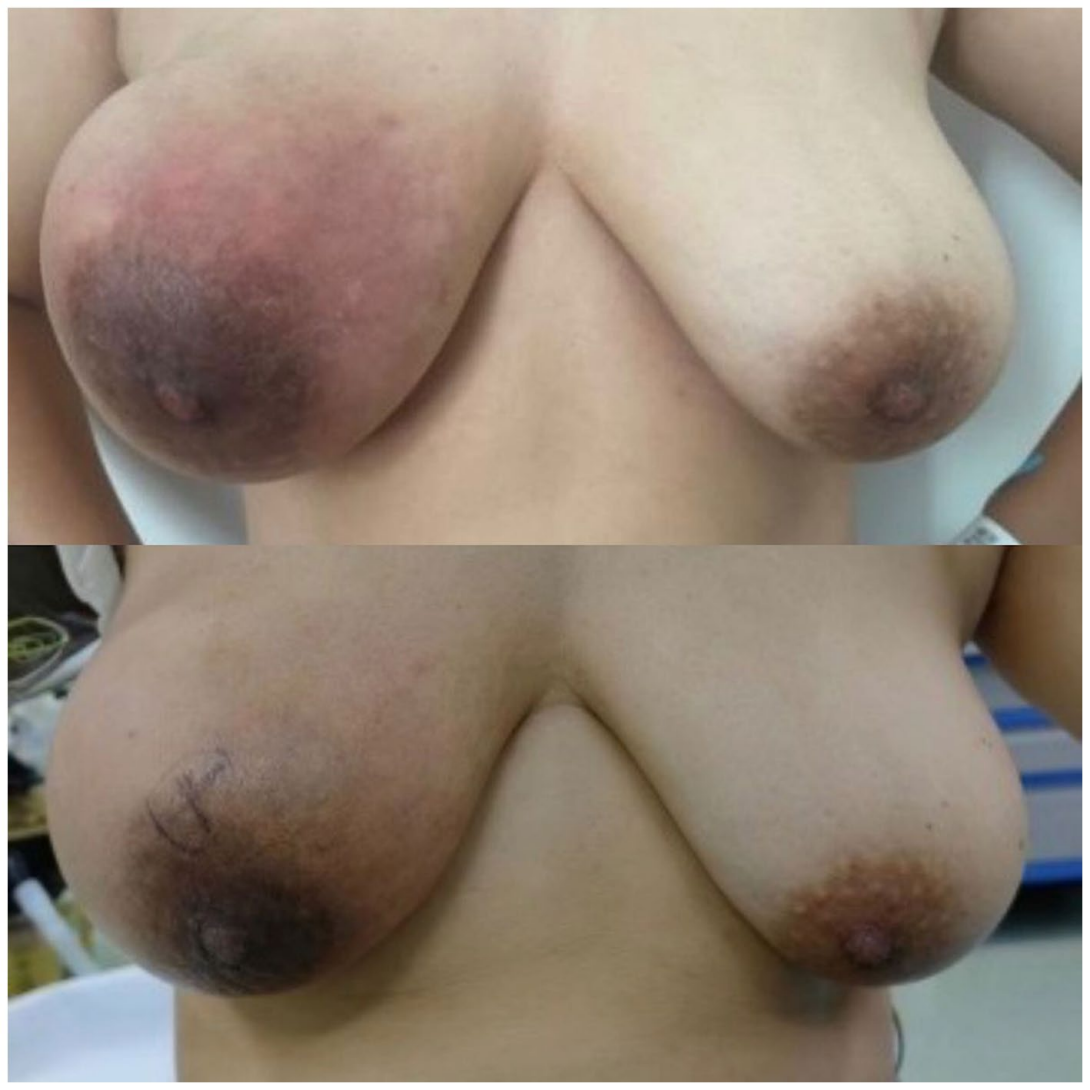
The photograph of the patient’s breast without any intervention treatment. The affected breast exhibits surface redness, mild elevation in skin temperature, and an increase in bloody discharge compared to before. The photograph of the breast on the day of surgery. After undergoing anti-inflammatory treatment, antifungal therapy, and corticosteroids, the breast mass has significantly reduced in size.

**Figure 5:**
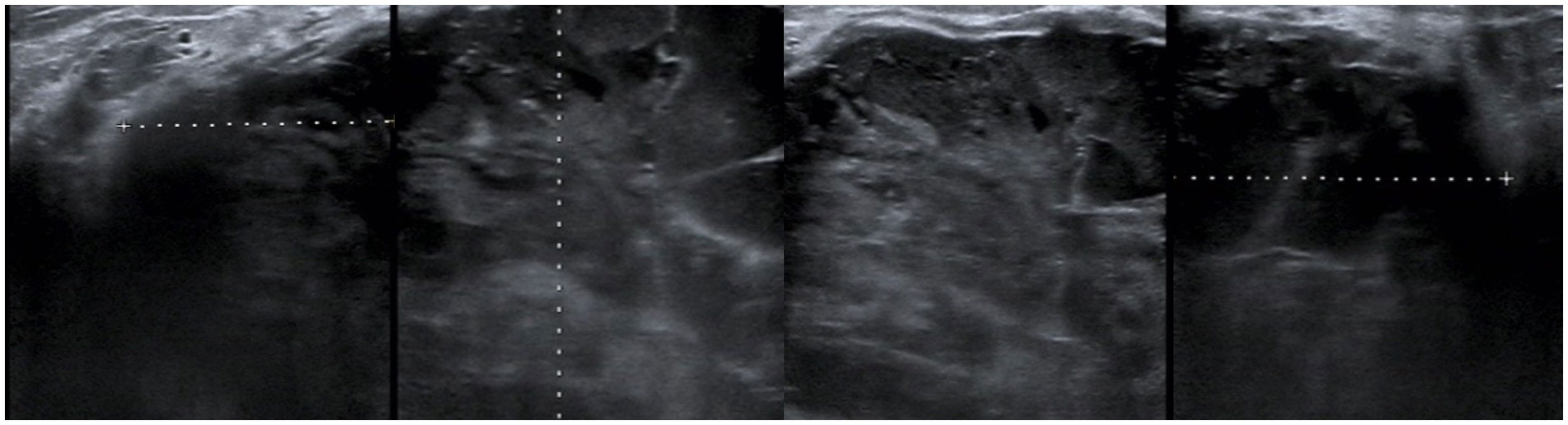
Ultrasound image from September 15, 2022. A hypoechoic mass measuring 154.2*66.1 mm is visible in the right breast, with indistinct borders and an irregular shape.

The patient had previously undergone a core needle biopsy at our hospital on June 21, 2022, which suggested a possible lymphoid malignancy. Further consultation at Peking University Third Hospital also indicated infiltration by a hematopoietic malignancy, but a definitive diagnosis could not be established. Subsequent immunohistochemical analysis at our hospital suggested a malignant tumor, recommending further immunohistochemical testing. However, the patient did not pursue further treatment. The uncertainty of her condition caused her significant anxiety. The mass rapidly enlarged, with increased redness, warmth, and pain in the breast and an increase in bloody nipple discharge (Figure 4). Immunohistochemical analysis on August 2, 2022, suggested Hodgkin’s lymphoma with infiltrating breast cancer, but the diagnosis remained unclear. Follow-up ultrasound on September 15, 2022, showed that the mass had grown to 154.2 × 66.1 mm (Figure 5). Further consultations at Tianjin Cancer Hospital and Capital Medical University led to a diagnosis of non-lymphoid malignancy with significant inflammatory cell reaction, but the cell origin remained uncertain. The ongoing uncertainty and physical symptoms took a toll on her emotional well-being. On September 21, 2025, PET-CT showed high metabolic activity in the right breast mass and right axillary lymph nodes, suggesting malignancy and possible metastasis. In order to rule out hematological and hereditary diseases, a series of tests were carried out, and the results are as follows (based on the date of the report publication). On September 22, 2022, bone marrow smear examination showed no obvious morphological abnormalities of bone marrow nucleated cells or abnormal cells. On September 23, immunophenotyping analysis of lymphocytes revealed a reduced proportion of lymphocytes, no obvious abnormalities in phenotype, an increased proportion of granulocytes at the developmental stage, and no significant abnormal primitive cells or monoclonal B-lymphocytes. On September 26, bone marrow biopsy indicated markedly active bone marrow hyperplasia in granulocytic, erythrocytic, and megakaryocytic lineages. On September 28, genetic testing for 26 genes associated with hereditary breast/ovarian cancer found no pathogenic or likely pathogenic mutations linked to hereditary cancer risk. On October 9, high-resolution chromosome analysis of 20 cells showed a normal karyotype.

Given the complexity of the case, a multidisciplinary team (MDT) comprising breast surgeons, oncologists, pathologists, and infectious disease specialists was convened. The MDT recommended anti-inflammatory treatment, bone marrow biopsy, and axillary lymph node excision. The patient received cefuroxime sodium (1.0 g daily) for 6 days, after which her infection markers and fever resolved. Bone marrow biopsy and flow cytometry showed no significant abnormalities. An excisional biopsy of the axillary lymph node revealed reactive lymphoid hyperplasia without metastasis on September 23, 2022. Despite these efforts, the diagnosis remained unclear. The lack of a clear diagnosis led to feelings of frustration and helplessness. The MDT recommended continued anti-inflammatory treatment, antifungal therapy, and corticosteroids. After treatment with linezolid (600 mg every 12 h) and dexamethasone (10 mg daily), the patient’s breast redness and pain significantly improved, the mass shrank, and the bloody discharge ceased. The improvement in her symptoms provided some relief and hope.

**Figure 6:**
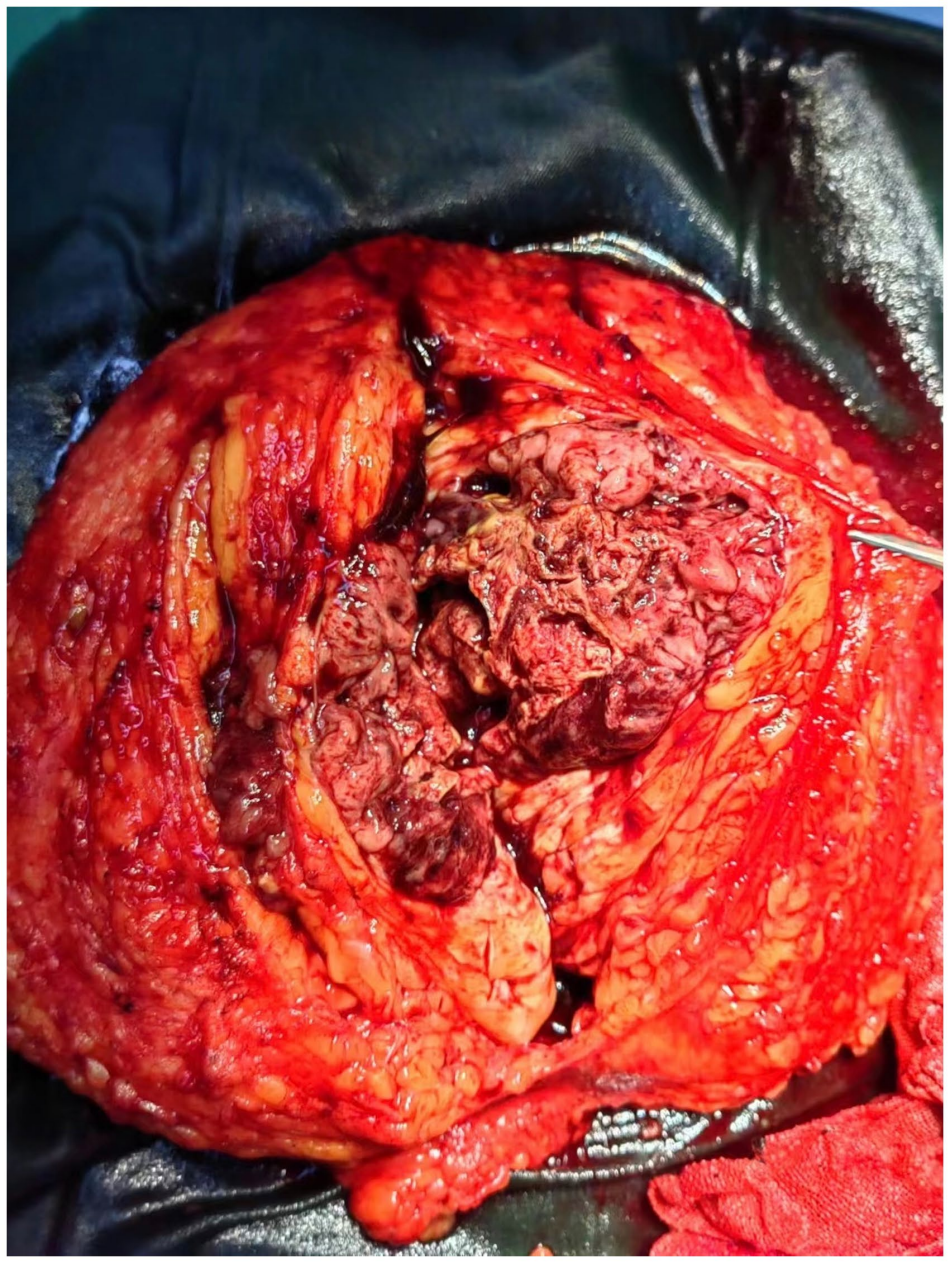
A tumor measuring approximately 80 mm*75 mm*75 mm is located in the central region of the breast and involves all quadrants, accompanied by severe hemorrhage and necrosis.

With informed consent, the patient underwent a right mastectomy on October 13, 2022 (Figure 4). The tumor measured approximately 80 mm × 75 mm × 75 mm, centrally located with extensive necrosis and hemorrhage (Figure 6). Postoperative pathology confirmed carcinosarcoma, with 5% high-grade infiltrating ductal carcinoma and 95% undifferentiated sarcoma. Immunohistochemical results included Ki-67 (60% in carcinoma, 40% in carcinosarcoma), ER (−), PR (−), Her-2 (0), AR (−), GATA3 (+ in carcinoma, − in carcinosarcoma), CKpan (+ in carcinoma, − in carcinosarcoma), CK5/6 (+ in carcinoma, − in carcinosarcoma), CK7 (+ in carcinoma, − in carcinosarcoma), E-Cadherin (+ in carcinoma, − in carcinosarcoma), Vimentin (− in carcinoma, + in carcinosarcoma), p63 (+ in carcinoma, − in carcinosarcoma). Given the complexity of the case, the postoperative pathology was sent for consultation to Fudan University Cancer Hospital, which confirmed metaplastic carcinoma with squamous differentiation and undifferentiated carcinosarcoma components. The confirmation of the diagnosis brought a sense of relief, despite the challenges ahead.

## Results

**Figure 7:**
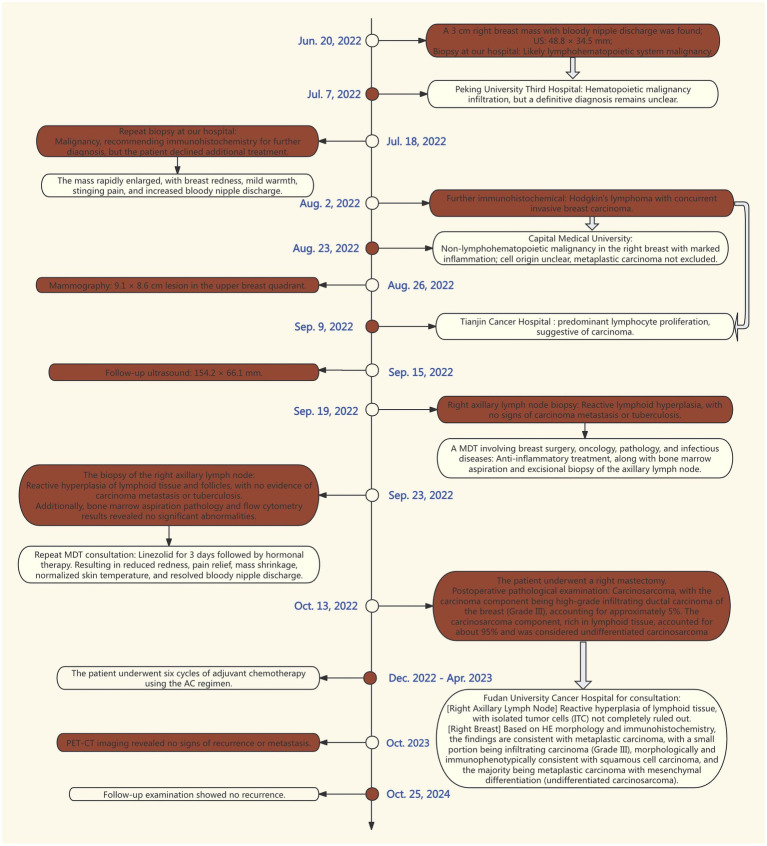
Outline the timeline of the patient’s medical visits. Each phase of intervention and treatment from June 20, 2022, to October 25, 2024, is depicted in the image. The symbol “
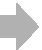
” indicates that the same pathology was sent to another hospital for consultation.

The patient subsequently received six cycles of AC chemotherapy (liposomal doxorubicin 50 mg every 3 weeks, Ifosfamide total 15 and 3 g daily for 5 days) and radiotherapy. A follow-up PET-CT in October 2023 showed no signs of recurrence or metastasis, and no evidence of recurrence was found on a follow-up examination on October 25, 2024. The above treatment process is shown in Figure 7.

## Discussion

Carcinosarcoma is a highly aggressive and rare malignant tumor of the breast, characterized by both carcinomatous and sarcomatous pathological features. This neoplasm is distinguished by its unique composition, featuring both epithelial and mesenchymal tumor elements, which significantly complicates preoperative diagnostic efforts. It has been recognized by the World Health Organization (WHO) as a distinct clinicopathological entity (1). In the 2003 WHO classification of breast tumors, carcinosarcoma was categorized as a subtype of metaplastic carcinoma of the breast (MCB) (2). The disease typically presents in women aged 32–77 years, with a peak incidence around 50 years of age (3). However, the youngest reported case of breast carcinosarcoma was a 24-year-old patient (4). Therefore, in clinical practice, the possibility of carcinosarcoma should not be overlooked when encountering young patients with breast tumors that remain diagnostically challenging despite extensive investigations.

Breast carcinosarcoma often mimics non-special type invasive breast carcinoma, typically presenting as an irregular, poorly mobile breast mass. It may also be accompanied by signs such as bloody nipple discharge or skin ulceration, resembling inflammatory breast cancer. However, its clinical manifestations are nonspecific and vary depending on the proportion of carcinomatous and sarcomatous components. Tumors predominantly composed of carcinoma may present with ipsilateral axillary or supraclavicular lymphadenopathy on physical examination or imaging. In contrast, tumors with a predominant sarcomatous component often exhibit rapid local growth and a higher likelihood of hematogenous rather than lymphatic metastasis. In this case, the patient’s right breast showed rapid enlargement due to tumor proliferation, and postoperative pathology confirmed a predominance of sarcomatous components, aligning with the clinical presentation. This underscores the importance of considering carcinosarcoma in the differential diagnosis when a breast mass demonstrates rapid growth accompanied by ductal or cutaneous invasion.

Differential diagnosis of carcinosarcoma is equally important. Breast carcinosarcoma should be distinguished from metaplastic carcinoma, including spindle cell carcinoma, carcinoma with cartilaginous and osseous metaplasia, matrix - producing carcinoma, fibrosarcoma, and other types of sarcoma. The key distinction lies in the fact that in carcinosarcoma, there is no transitional zone between the carcinoma component and the sarcoma- like component. In contrast, metaplastic carcinoma exhibits a distinct transitional zone (5, 6). Phyllodes tumors are breast tumors composed of stromal and epithelial components. Based on stromal cellularity, atypia, and mitotic activity, they are classified into benign, borderline, and malignant types. The stromal component of malignant phyllodes tumors exhibits sarcoma-like features. Some researchers suggest that when the epithelial component of malignant phyllodes tumors becomes malignant, it can be considered a subtype of true carcinosarcoma (6). However, others argue that if non-invasive carcinoma is present in the stromal component of malignant phyllodes tumors, it should be reported separately in pathological reports instead of being classified as carcinosarcoma (7, 8).

Ultrasonography, as the most commonly used auxiliary examination, often reveals round or oval hypoechoic masses with heterogeneous echogenicity, some of which may exhibit lobulated contours and posterior acoustic enhancement (9). The heterogeneity in echogenicity is one of the characteristic features of breast carcinosarcoma, attributed to the uneven proportion of carcinomatous and sarcomatous components (10). On mammography, these tumors typically present as high-density masses with indistinct margins, and calcifications, when present, are usually amorphous or clustered (9). Notably, the clinical manifestations, ultrasonographic, and mammographic features of breast carcinosarcoma closely resemble those of non-special type invasive breast carcinoma, often leading to diagnostic uncertainty or misdiagnosis. Definitive diagnosis relies on pathological examination; however, in our case, despite two preoperative needle biopsies and consultations at multiple authoritative institutions, a conclusive diagnosis remained elusive, highlighting the tumor’s heterogeneity and diagnostic complexity.

Additionally, this patient experienced intermittent fever, which resolved with intravenous antibiotics, mimicking inflammatory breast cancer or hematologic disorders. This underscores that breast carcinosarcoma may present with inflammatory-like clinical features, particularly in patients with intermittent fever. Clinicians should carefully evaluate patient history and imaging findings to avoid misdiagnosis. Pathological examination and advanced imaging can help differentiate inflammatory breast cancer. Bone marrow biopsy can exclude blood - system diseases, as done in this patient’s case. For patients presenting with unexplained fever and localized masses, a high index of suspicion and prompt pathological evaluation are essential.

The treatment approach for breast carcinosarcoma does not significantly differ from that of breast carcinoma. Studies have shown that breast-conserving surgery (BCS) combined with radiotherapy has been primarily validated as safe and effective compared to mastectomy (11). Therefore, breast-conserving surgery is the preferred option for breast carcinosarcoma, provided that negative margins can be achieved. However, if the tumor-to-breast volume ratio is disproportionate, resulting in unsatisfactory cosmetic outcomes, or if the disease is advanced, mastectomy is recommended (9). Furthermore, axillary lymph node metastasis is less common in carcinosarcoma compared to other breast cancer types [approximately 20% (3)]. The necessity of sentinel lymph node biopsy remains unclear in current guidelines and should be determined based on tumor size, grade, and imaging evidence of lymphatic infiltration. However, if clinical evaluation is positive, axillary lymph node dissection is still warranted. Postoperative adjuvant therapy is essential, and studies by Hennessy et al. (12) suggest that anthracycline-based chemotherapy regimens yield superior outcomes compared to traditional CMF regimens in breast carcinosarcoma patients.

Due to its highly aggressive nature and frequent triple-negative immunophenotype, breast carcinosarcoma shares the high risk of recurrence, metastasis, and poor prognosis characteristic of triple-negative breast cancer. Common sites of metastasis include the lungs, brain, bones, and liver, and the prognosis for these patients is generally poor (3). A survival analysis of 47 carcinosarcoma patients revealed that tumor size, treatment modality, time from symptom onset to diagnosis (TI), and adjuvant chemotherapy significantly influenced disease-free survival (DFS) and overall survival (OS). Multivariate analysis further confirmed that TI and adjuvant chemotherapy were independent prognostic factors for DFS, while TI, N-stage, and adjuvant chemotherapy were independent prognostic factors for OS. The 5-year DFS and OS rates were approximately 56.3 and 72.2%, respectively (13). Another study reported 5-year survival rates of 73, 59, 44, and 0% for clinical stages I, II, III, and IV, respectively (14). Therefore, early detection and treatment are critical and may significantly impact the prognosis of carcinosarcoma.

## Conclusion

Overall, the nonspecific clinical presentation of breast carcinosarcoma makes a definitive preoperative diagnosis challenging, and even preoperative pathological examination may fail to provide clarity. This leaves clinicians at a loss when it comes to formulating the next steps in treatment. Therefore, when encountering similar cases, clinicians should meticulously analyze the available “clues,” such as rapidly enlarging masses over a short period, accompanied by skin and ductal invasion, as well as ultrasonographic findings of irregularly shaped masses with heterogeneous echoes. These signs should raise suspicion for the possibility of carcinosarcoma. Surgery remains the primary treatment modality for carcinosarcoma, and postoperative adjuvant chemotherapy, in combination with radiotherapy, may improve the prognosis for patients with this aggressive malignancy.

## Data Availability

The raw data supporting the conclusions of this article will be made available by the authors, without undue reservation.
